# Seroprevalence and Temporal Trends of Syphilis Among Blood Donors at Dhiraj Blood Bank in Waghodia, Gujarat, India: A Retrospective Study

**DOI:** 10.7759/cureus.116628

**Published:** 2026-09-21

**Authors:** Devanshi Gosai, Falak Modi, Nisha J Parmar, Khusbu Chhaya

**Affiliations:** 1 Pathology, Smt. B.K. Shah Medical Institute and Research Centre, Sumandeep Vidyapeeth Deemed to Be University, Piparia, IND; 2 Microbiology, Prasad Medical College and Hospital, Unnao, IND; 3 Pathology, Dr. N.D. Desai Medical College, Dharamsinh Desai University, Nadiad, IND

**Keywords:** blood donors, blood safety, enzyme-linked immunosorbent assay, seroprevalence, syphilis, transfusion-transmitted infections, venereal disease research laboratory test

## Abstract

Background: Syphilis remains an important transfusion-transmitted infection (TTI) despite improvements in donor selection and blood screening practices. Continuous surveillance among blood donors is essential for ensuring transfusion safety and monitoring disease trends.

Objective: To determine the seroprevalence and temporal trends of syphilis among blood donors at a tertiary care blood bank in Gujarat, India.

Materials and Method: A retrospective, record-based cross-sectional study was conducted at Dhiraj Blood Bank, Dhiraj General Hospital, Waghodia, Gujarat, India. Blood donor records from January 2023 to July 2025 were reviewed. All eligible voluntary and replacement blood donors who underwent routine screening were included. Initial screening for syphilis was performed using the Venereal Disease Research Laboratory test (VDRL), and reactive samples were confirmed using Syphi Microlisa, a qualitative enzyme-linked immunosorbent assay (ELISA) for the detection of antibodies to *Treponema pallidum*. Data were analyzed using descriptive statistics, and seroprevalence was expressed as percentages.

Results: A total of 7,471 blood donations were screened during the study period. 111 (1.49%) samples were reactive by VDRL, of which 45 (40.5%) were reactive on Syphi Microlisa, yielding an overall syphilis seroprevalence of 0.60%. The annual seroprevalence was 0.85% in 2023, 0.08% in 2024, and 0.90% in 2025 (up to July), showed variation across the study years. More than half of the VDRL-reactive samples were non-reactive on Syphi Microlisa, highlighting the importance of appropriate second-step treponemal testing.

Conclusion: Syphilis continues to be detected among apparently healthy blood donors, emphasizing the need for sustained donor screening and surveillance. The combined use of VDRL and confirmatory ELISA improves diagnostic accuracy and supports safe transfusion practices. Regular monitoring of seroprevalence trends and strengthening blood safety measures remain essential for preventing TTIs.

## Introduction

Blood transfusion is an indispensable component of modern healthcare, providing lifesaving support during trauma, major surgical procedures, obstetric emergencies, hematological disorders, and various medical illnesses. Despite stringent donor selection criteria and advances in laboratory screening, transfusion-transmitted infections (TTIs) remain a significant concern for blood safety. Sharma et al. reported that routine screening of blood donors is essential for minimizing the transmission of infectious diseases and ensuring the safety of the blood supply in tertiary care settings in India [1].

In a study conducted in western India, Shah et al. observed that syphilis continues to be detected among apparently healthy blood donors despite its relatively low prevalence, highlighting the continued need for mandatory screening of every donated blood unit [2]. Similarly, Singhal et al. demonstrated that syphilis seropositivity among blood donors is often associated with asymptomatic infection and emphasized the importance of serological screening for identifying infected individuals who would otherwise remain undetected [3].

Paudel et al. reported a syphilis seroprevalence of 0.16% among blood donors in Puducherry, indicating that transfusion-transmitted syphilis remains a relevant public health concern despite its low occurrence [4]. Jain et al. further demonstrated that syphilis-positive donors may also harbor other transfusion-transmissible infections, emphasizing the importance of comprehensive donor screening for multiple pathogens [5]. In northern Karnataka, seroprevalence of 0.12% was found among clinically healthy blood donors and concluded that continuous surveillance is essential for maintaining blood safety [6]. Fluctuations in syphilis were prevalent before and after the COVID-19 pandemic in western Rajasthan, suggesting that epidemiological trends may vary over time and require ongoing monitoring [7]. A three-year retrospective study from Himachal Pradesh, observed consistently low seroprevalence but recommended sustained surveillance to detect changing trends in TTIs among blood donors [8].

A systematic review and meta-analysis by Golia et al. demonstrated that although syphilis constitutes a relatively small proportion of TTIs in India, it remains an important marker for monitoring blood safety and evaluating the effectiveness of donor screening programs [9].

International studies have reported considerable variation in syphilis seroprevalence among blood donors. Wu et al. found that increasing age was significantly associated with higher syphilis seropositivity among blood donors in southern China [10]. Djimadoum et al. reported that syphilis remained one of the major TTIs among blood donors in Chad, underscoring the need for rigorous donor screening in resource-limited settings [11]. Similarly, Arshad et al. documented persistent syphilis seroreactivity among apparently healthy blood donors in Pakistan and recommended strict implementation of screening protocols to prevent transfusion-associated transmission [12].

The World Health Organization, in its Global Status Report on Blood Safety and Availability, recommends universal screening of all donated blood for syphilis along with other major TTIs as a fundamental strategy for ensuring safe blood transfusion services worldwide [13].

Assessment of the seroprevalence and temporal trends of syphilis among blood donors not only protects blood recipients but also serves as an important indicator of infection patterns within the healthy adult population. Such information supports evaluation of donor selection policies, strengthens blood safety programs, and guides targeted public health interventions. Therefore, the present study was undertaken to determine the seroprevalence and trends of syphilis among blood donors at a tertiary care blood bank.

## Materials and methods

Study design

This retrospective, observational, record-based cross-sectional study was conducted by reviewing blood donor screening records maintained at Dhiraj Blood Bank in Waghodia, Gujarat, India. The study was designed to determine the seroprevalence and temporal trends of syphilis among blood donors over the study period using routinely collected laboratory screening data.

Study setting

The study was carried out at Dhiraj Blood Bank, attached to Dhiraj General Hospital. The blood bank functions under the Department of Pathology, Immunohematology and Blood Transfusion and provides transfusion services to four districts of central Gujarat. All donated blood units undergo mandatory screening for TTIs in accordance with the standard operating procedures of the blood bank and the guidelines of the National Blood Transfusion Council (NBTC)/National AIDS Control Organisation (NACO) [14].

Study duration

The study included all eligible blood donations received between January 2023 and July 2025, covering a period of 31 months. Data were analyzed year-wise to compare the trends observed during 2023, 2024, and the partial year 2025.

Study population

The study population comprised all voluntary and replacement blood donors who donated blood at Dhiraj Blood Bank during the study period. Each donated unit underwent routine serological screening for TTIs before being considered suitable for clinical use.

Inclusion criteria

All blood donations collected and screened between January 2023 and July 2025 were included in the study. Donors fulfilled the standard eligibility criteria for blood donation as prescribed by NBTC/NACO, including age, body weight, hemoglobin level, and general health status.

Exclusion criteria

Blood donations with incomplete laboratory records or those discarded before serological testing because of inadequate collection, clotting, or other technical reasons were excluded from the analysis.

Sample size

A complete enumeration of all eligible blood donations received during the study period was undertaken. Since the study included all available donor records, no formal sample size calculation was performed. A total of 7,471 blood donations were included for analysis. The year wise distribution of blood donations is shown in Table 1

**Table 1:** Annual blood bag collection at the blood bank during the study period (2023–2025) *2025 data represent blood bags collected from January through July 2025 only and do not reflect the complete calendar year. Values are presented as the number of blood bags collected. A total of 7,471 blood bags were collected during the study period.

Year	Blood bags collected
2023	2,714
2024	2,553
2025*	2,204
Total	7,471

Screening procedure

All donated blood units were screened for syphilis according to the standard operating procedure followed at Dhiraj Blood Bank. Initial screening was performed using the R.P.R. (Venereal Disease Research Laboratory test (VDRL)) Kit (Cat. No. IS 6240) (Bio Lab Diagnostics (I) Pvt. Ltd., India). The assay was performed as a qualitative slide test in accordance with the manufacturer's instructions and the blood bank's standard operating procedure [15]. A reactive result was defined by the presence of visible flocculation/clumping of carbon particles, while absence of visible flocculation was interpreted as non-reactive [15].

Samples reactive on VDRL screening were subsequently tested using Syphi Microlisa, a qualitative enzyme-linked immunosorbent assay (ELISA) for detecting antibodies to *Treponema pallidum*. The kit was manufactured by J. Mitra & Co. Pvt. Ltd., New Delhi, India (Cat. No. IR012096) [16]. The assay was performed according to the manufacturer's instructions and the blood bank's standard operating procedure. The reaction was read at 450 nm with a 630-nm reference filter [16]. The cut-off value (COV) was calculated as the mean absorbance of the negative control plus 0.2. The sample-to-cut-off ratio (S/CO) was calculated as the sample absorbance divided by the COV. Samples with an S/CO <1.0 were considered non-reactive, whereas samples with an S/CO ≥1.0 were considered reactive [16]. Samples with S/CO between 0.9 and 1.1 were initially considered reactive and retested in duplicate according to the manufacturer's instructions [16].

The assay run was considered valid when the mean negative-control absorbance was ≤0.100 and the positive-control absorbance was ≥1.5 [16]. Appropriate controls were included in accordance with the manufacturer's instructions. Only samples reactive on VDRL screening and subsequently confirmed positive by Syphi Microlisa were classified as syphilis-positive for the final analysis. VDRL-reactive samples with negative Syphi Microlisa results were not classified as confirmed syphilis cases.

Data collection

Data were retrieved retrospectively from the blood bank donor registers and laboratory screening records. The information collected included donor age, sex, donor type, total number of blood donations, number of VDRL-reactive samples, and number of Syphi Microlisa-reactive samples. Data were anonymized before analysis to ensure donor confidentiality.

Donor selection and pre-donation assessment

Blood donors undergo routine donor selection and pre-donation assessment before collection, including evaluation of general health and eligibility in accordance with applicable blood donation criteria. The donor history questionnaire is intended to identify medical conditions, recent illnesses, medication use, previous transfusion or donation history, and other conditions or exposures that may warrant temporary or permanent deferral. Nevertheless, questionnaire-based screening depends on information provided by donors and may not detect asymptomatic infections. Consequently, laboratory screening remains an essential independent component of blood safety.

Donor notification and counseling

Donors with confirmed reactive results require confidential notification and appropriate counseling regarding the significance of the test result. The donor should be advised that a reactive screening result does not necessarily establish active infection when a confirmatory test is negative, whereas confirmed seroreactivity warrants appropriate clinical evaluation and referral for further management. Counseling should include information regarding the need for medical evaluation, prevention of potential transmission, and follow-up according to institutional protocols. Maintaining confidentiality throughout notification and referral is essential to protect donor privacy and reduce stigma.

Statistical analysis

The collected data were entered into Microsoft Excel 2019 for compilation and analysis. Overall and subgroup-specific syphilis seroprevalence were calculated as the number of Syphi Microlisa-reactive samples divided by the corresponding total number of blood donations, multiplied by 100. Annual seroprevalence of syphilis was calculated using the following formula:

$\text{Seroprevalence (\%)} = \frac{\text{Number of ELISA-confirmed syphilis-positive donations}}{\text{Total blood donations}} \times 100$

The results were expressed as frequencies and percentages and presented in tables and figures. Year-wise and demographic subgroup variations were assessed descriptively. No inferential statistical tests were performed, as the primary analysis was descriptive.

Ethical considerations

Prior approval for the study was obtained from the Institutional Ethics Committee (SVIEC/ON/Medi/RP/July/25/103). The study utilized anonymized, retrospective blood bank records without accessing personal identifiers. Confidentiality of donor information was strictly maintained throughout the study, and the research was conducted in accordance with the ethical principles governing record-based biomedical research.

## Results

Table 2 presents the year-wise distribution of blood donations, VDRL-reactive samples, and ELISA-confirmed syphilis-positive cases. A total of 7,471 blood donations were screened during the study period. Among these, 111 (1.49%) samples were reactive on initial VDRL screening, while 45 (0.60%) were confirmed positive for syphilis by Syphi Microlisa. The annual seroprevalence of confirmed syphilis was 0.85% (23 out of 2,714) in 2023, decreased markedly to 0.08% (2 out of 2,553) in 2024, and increased to 0.90% (20 out of 2,204) during January to July 2025. The lowest seroprevalence was observed in 2024, whereas the highest observed seroprevalence during the study period was recorded in the available 2025 data (January-July).

**Table 2:** Year-wise distribution of VDRL-reactive and ELISA-confirmed syphilis cases among blood donors (2023–2025) *2025 data represent donations collected from January through July 2025 only and do not reflect the complete calendar year. Values are presented as number (%), with percentages calculated using the total blood donations for the corresponding year as the denominator. VDRL: Venereal Disease Research Laboratory test; ELISA: Enzyme-linked immunosorbent assay

Year	Total blood donations	VDRL reactive, n (%)	ELISA confirmed positive, n (%)
2023	2,714	63 (2.32)	23 (0.85)
2024	2,553	5 (0.20)	2 (0.08)
2025*	2,204	43 (1.95)	20 (0.90)
Total	7,471	111 (1.49)	45 (0.60)

The demographic characteristics and distribution of VDRL- and Syphi Microlisa-reactive samples are presented in Table 3. Among 7,471 donors, most were aged 25-34 years (32.4%), followed by 35-44 years (27.9%). VDRL reactivity ranged from 1.04% to 1.67%, while Syphi Microlisa reactivity ranged from 0.37% to 0.72%, with the highest prevalence among donors aged 35-44 years (0.72%). Most donors were male (89.1%), with Syphi Microlisa reactivity of 0.62% versus 0.49% among females.

**Table 3:** Demographic characteristics and distribution of VDRL reactivity and Syphi Microlisa reactivity among blood donors Demographic distribution of blood donors according to age group and gender with corresponding VDRL reactivity and Syphi Microlisa reactivity. Seroprevalence was calculated as the number of Syphi Microlisa-reactive donors divided by the total number of donors within each category × 100. Values are presented as n (%) where indicated. VDRL: Venereal Disease Research Laboratory test

Characteristic	Total donors, n (%)	VDRL reactive, n (%)	Syphi Microlisa reactive, n (%)	Seroprevalence (%)
Age group				
<25 years	1,345 (18.0)	14 (1.04)	5 (0.37)	0.37
25–34 years	2,418 (32.4)	36 (1.49)	14 (0.58)	0.58
35–44 years	2,087 (27.9)	34 (1.63)	15 (0.72)	0.72
≥45 years	1,621 (21.7)	27 (1.67)	11 (0.68)	0.68
Sex				
Male	6,653 (89.1)	101 (1.52)	41 (0.62)	0.62
Female	818 (10.9)	10 (1.22)	4 (0.49)	0.49
Total	7,471 (100.0)	111 (1.49)	45 (0.60)	0.6

Table 4 shows the results of confirmatory ELISA testing among the 111 VDRL-reactive blood donor samples. Among the 111 VDRL-reactive samples, 45 (40.5%) were reactive on Syphi Microlisa, whereas 66 (59.5%) were non-reactive. Thus, 59.5% of VDRL-reactive samples were not reactive on the second-step ELISA, highlighting the importance of appropriate treponemal testing in interpreting non-treponemal screening results.

**Table 4:** Syphi Microlisa reactivity among VDRL-reactive blood donor samples Values are presented as number (%). Percentages were calculated using the total number of VDRL-reactive samples (n = 111) as the denominator. Syphi Microlisa non-reactive samples represent VDRL-reactive samples that were non-reactive on the second-step ELISA. VDRL: Venereal Disease Research Laboratory test; ELISA: Enzyme-linked immunosorbent assay

VDRL-reactive samples (n=111)	Number	Percentage (%)
Syphi Microlisa reactive	45	40.5
Syphi Microlisa non-reactive	66	59.5
Total	111	100

## Discussion

The present study demonstrated an overall syphilis seroprevalence of 0.60% among 7,471 blood donors. This prevalence is identical to that reported by Paudel et al. from Puducherry, who also observed a seroprevalence of 0.60%, suggesting a comparable burden of syphilis among blood donors in tertiary care settings [4]. Similarly, Patel NH et al. reported a prevalence of approximately 0.50% among blood donors in Nadiad, Gujarat [17], which is comparable to the findings of the present study. Patel DD et al. reported a slightly lower seroprevalence of 0.40% among blood donors in Ahmedabad [18], indicating regional variation within Gujarat. A retrospective study from western India involving over 26,000 blood donations also demonstrated a comparable seroprevalence of 0.49% [19], which is comparable with the findings of the present study.

The comparison of the present study with similar Indian studies is presented in Table 5.

**Table 5:** Comparison of syphilis seroprevalence among blood donors between the present study and previously published Indian studies The table compares the seroprevalence of ELISA-confirmed syphilis among blood donors reported in the present study with findings from selected studies conducted in different regions of India. Seroprevalence is expressed as a percentage of all blood donors screened. The study periods are presented as reported in the respective publications. ELISA: Enzyme-linked immunosorbent assay

Study	Study area	Study period	Syphilis seroprevalence (%)
Present study	Waghodia, Gujarat	Jan 2023–Jul 2025	0.60
Paudel et al. [4]	Puducherry	2021–2023	0.60
Patel NH et al. [17]	Nadiad, Gujarat	Retrospective	0.50
Patel DD et al. [18]	Ahmedabad, Gujarat	Retrospective	0.40
Mallya et al. [19]	Gujarat/Maharashtra	Jan 2020–Sep 2021	0.49
Kulkarni et al. [6]	Northern Karnataka	Retrospective	0.21

Although the observed seroprevalence is at the higher end of the range reported from Indian blood banks, it remains within the expected epidemiological pattern. Kulkarni et al. documented a lower prevalence of 0.21% among healthy blood donors in northern Karnataka, highlighting geographical differences in disease burden [6]. Nandkishore et al., in a multicenter retrospective analysis, also reported considerable regional variation in TTIs across India, emphasizing the influence of donor characteristics and local epidemiology [20].

Year-wise analysis revealed a decline in seroprevalence from 0.85% in 2023 to 0.08% in 2024, followed by an increase to 0.90% in 2025. A similar fluctuation in syphilis prevalence before and after the COVID-19 pandemic was reported by Bharti et al. in western Rajasthan [7]. Likewise, Arora et al. observed changing annual trends in TTIs among blood donors in Himachal Pradesh, supporting the need for continuous surveillance [8].

Among the 111 VDRL-reactive samples in the present study, 45 (40.5%) were reactive on Syphi Microlisa. Kakkar et al. demonstrated that ELISA possesses superior specificity compared with non-treponemal tests such as Rapid Plasma Reagin test (RPR)/VDRL, thereby reducing false-positive results [21]. Similarly, Golia et al., in their systematic review and meta-analysis, concluded that confirmatory treponemal assays improve the accuracy of blood donor screening programs [9].

International studies have also reported low but persistent syphilis seroprevalence among blood donors. Wu et al. found that increasing age was significantly associated with higher syphilis seropositivity among blood donors in Southern China [10]. Djimadoum et al. reported that syphilis remains an important transfusion-transmissible infection among blood donors in Chad despite routine screening [11]. Arshad et al. similarly observed persistent syphilis seroreactivity among apparently healthy blood donors in Pakistan and emphasized the need for strict donor screening [12].

The World Health Organization continues to recommend universal screening of all donated blood for syphilis as an essential component of blood safety programs worldwide [13]. The findings of the present study are consistent with recommendations for continued surveillance, rigorous donor selection, confirmatory testing of reactive samples, and periodic review of blood donor screening data to ensure a safe and adequate blood supply.

Possible reasons for seropositivity among blood donors

The detection of syphilis seropositivity among apparently healthy blood donors may reflect the asymptomatic or clinically unrecognized nature of infection. Blood donors undergo routine health assessment before donation; however, individuals with early, latent, or previously unrecognized syphilis may not have clinically apparent symptoms at the time of donation. In addition, donor selection procedures are primarily intended to identify individuals who are medically suitable for donation and cannot completely exclude all persons with asymptomatic infections. The presence of confirmed seropositivity therefore reinforces the importance of laboratory-based screening in addition to clinical donor assessment.

However, the present study did not collect detailed behavioral or clinical risk-factor information, and therefore no specific behavioral or epidemiological factor can be identified as responsible for the observed seropositivity. The observed findings should consequently be interpreted as evidence of detectable serological infection among the screened donor population rather than as an assessment of individual risk factors for syphilis.

Limitations

This study has several limitations. First, it was conducted at a single tertiary care blood bank, which may limit the generalizability of the findings to other regions and donor populations in India. Second, the retrospective design relied on routinely maintained blood bank records, limiting the availability of detailed demographic, behavioral, clinical, and epidemiological risk-factor information. Consequently, potential determinants of syphilis seropositivity could not be assessed.

Third, the study period extended only through July 2025; therefore, the 2025 findings represent January-July 2025 and should not be interpreted as a complete annual estimate or directly compared with complete calendar years without caution. The observed year-wise variation should be regarded as descriptive rather than evidence of a definitive epidemiological trend.

Fourth, VDRL-reactive samples were confirmed using Syphi Microlisa; additional treponemal confirmatory assays such as *Treponema pallidum* Hemagglutination Assay (TPHA) or fluorescent treponemal antibody absorption (FTA-ABS) were not available. Therefore, the classification of ELISA-confirmed samples may not be directly comparable with studies using different confirmatory algorithms. Finally, detailed behavioral and clinical risk-factor information was not available; therefore, the observed demographic differences could not be further evaluated in relation to behavioral or clinical determinants of syphilis seropositivity.

## Conclusions

The present study identified a low but persistent level of syphilis seropositivity among blood donors at a tertiary care blood bank in Gujarat. The substantial proportion of VDRL-reactive samples that were non-reactive on Syphi Microlisa highlights the importance of appropriate second-step treponemal testing. The observed year-wise variation should be interpreted cautiously because the 2025 data represent only January through July. Continued routine screening, appropriate testing, confidential donor notification and counseling, and systematic surveillance remain important components of blood safety. Further multicenter prospective studies with larger and more diverse donor populations, detailed demographic and behavioral information, and standardized treponemal confirmatory testing are warranted.
